# The Spinal Neurons Exhibit an ON-OFF and OFF-ON Firing Activity Around the Onset of Fictive Scratching Episodes in the Cat

**DOI:** 10.3389/fncel.2018.00068

**Published:** 2018-03-13

**Authors:** Carlos A. Cuellar, Braniff De La Torre Valdovinos, Nayeli Huidobro, Rodolfo Delgado-Lezama, Rafael Ornelas-Kobayashi, Elias Manjarrez

**Affiliations:** ^1^Department of Neurologic Surgery, Mayo Clinic, Rochester, MN, United States; ^2^Centro Universitario de Ciencias Exactas e Ingenierías, Universidad de Guadalajara, Guadalajara, Mexico; ^3^Instituto de Fisiología, Benemerita Universidad Autonoma de Puebla, Puebla, Mexico; ^4^Departamento de Fisiologia, Biofisica y Neurociencias, CINVESTAV IPN, Mexico City, Mexico; ^5^University of Groningen, Groningen, Netherlands

**Keywords:** CPG, spinal cord, fictive scratching, flexor phase, interneurons, firing rate, lognormal

## Abstract

In a previous report, we found neurons with ON-OFF and OFF-ON firing activity in the obex reticular formation during scratching. The aim of the present study was to examine whether the spinal neurons also exhibit this type of activity in relation to the “postural stage” of fictive scratching in the cat. We found that the extensor and intermediate scratching neurons exhibit an ON-OFF firing rate; conversely, the flexor neurons show an OFF-ON activity, relative to every scratching episode. These patterns of spiking activity are similar to those found in neurons from the obex reticular formation during scratching. Our findings provide support to the following hypotheses. First, there is a possible functional link between supraspinal and spinal, ON-OFF and OFF-ON neuronal groups. Second, the fictive goal-directed motor action to maintain the fictive “postural stage” of the hindlimb during fictive scratching is associated with the neuronal tonic activity of the OFF-ON spinal neurons, whereas the ON-OFF spinal neurons are associated with an extensor tone that occurred prior the postural stage.

## Introduction

In cats, the mechanical stimulation of receptive fields from the neck and the pinna produces scratching behavior. In 1906, Sherrington showed that the scratching is a stereotyped and relatively simple motor pattern that can be studied to provide insights into the organization of the spinal circuitry that generates rhythmic motor output (Sherrington, 1910). Subsequent studies showed that the scratching could be useful to study the spinal networks known as central pattern generators (CPGs) (Brown, 1911). Many stereotyped motor patterns are produced by the CPG (Grillner, 1981; Berkowitz and Stein, 1994; Whelan, 1996; Kiehn and Kjaerulff, 1998; Orlovsky et al., 1999; Kiehn, 2006). An advantage of the scratching is that it can be easily elicited as a fictive motor task in decerebrate cats, while the animals are paralyzed. In its broadest definition, the term “fictive” can be defined as the motor manifestation of physiological processes occurring in the nervous system in the absence of movement. An animal preparation that exhibits “fictive scratching” could be obtained after the section of motoneuron axons or when the muscles are removed or paralyzed. In general, many spinal interneurons and motoneurons are involved in this “fictive goal-directed motor action” of scratching. We will employ here the term “goal-directed motor action” because changes in tonic neuronal activity are related to “goal-directed behaviors” executed automatically in non-human primates (see Apicella et al., 1991, 1997, 2011; Ravel et al., 2001). To avoid confusions, we will employ the term “motor action” instead of “behavior.” The term fictive motor action of scratching has been employed previously in other studies of neuronal rhythmic activity in cats (Fedirchuk et al., 2013).

The scratching in the cat starts with a “goal-directed motor action,” when the active hindlimb reaches the surface of the skin and is maintained there. This first phase is commonly termed “postural stage” and is characterized by the tonic activation of flexor nerves (Deliagina et al., 1983). The second phase, or “rhythmical stage,” is performed when the hindlimb carries out rhythmic movements of alternation between flexor and extensor muscles at a frequency of 4 Hz approximately to scratch the skin, while the other limbs contribute supporting the animal position (Sherrington, 1910; Kuhta and Smith, 1990). Several studies have described rhythmically active neurons during scratching located in the cat spinal cord (Berkinblit et al., 1978a,b, 1980; Baev et al., 1981; Deliagina et al., 1983; Cuellar et al., 2009; Pérez et al., 2009). Although the firing frequency of some spinal neurons was studied and classified according to their firing activity in the cat (Berkinblit et al., 1978a,b; Baev et al., 1981) and subsequently in the turtle (Berkowitz and Stein, 1994; Berkowitz, 2001, 2005, 2008; Berkowitz et al., 2006; Guzulaitis et al., 2014), it was not until 2016 that Petersen and Berg (2016) described that the distribution of firing rates of populations of spinal neurons was lognormal. Moreover, Bayev and Kostyuk (1981) described a positive DC-shift during the tonic flexor phase, recorded on the dorsum of the spinal cord during fictive scratching. More recently, Pérez et al. (2009) and Cuellar et al. (2009) further analyzed the DC-shift and the sinusoidal potentials produced during fictive scratching, describing a phenomenon of “traveling electrical waves” produced by the rhythmic activation of spinal neurons. Interestingly, the electrical field potentials recorded as sinusoidal waves (sinusoidal-CDPs), “sweep” the lumbosacral spinal cord rostrocaudally from L4 to S1. Thus, Cuellar et al. (2009) claimed that the sinusoidal waves represent the combined activity of the CPG and associated elements described as “followers” and the activity of motoneurons (i.e., the output). Interestingly, the spinal positive DC-shift recorded on the surface of the cord dorsum did not exhibit any rostrocaudal propagation (Cuellar et al., 2009). In a subsequent study, Tapia et al. (2013) recorded a slow electrical potential in the bulbar brainstem close to the obex (OSP), which occurred 800 ms prior the onset of the spinal DC-shift associated to a scratching episode. In this report, Tapia et al. (2013) showed that in the region of the obex, there are neurons with an interesting tonic firing activity during the OSP. They found two classes of neurons in the obex region; one termed ON-OFF, and the other, OFF-ON, because their sustained activity decreased or increased during the OSP, respectively.

The purpose of the present study is to analyze peri-event firing rates of spinal neurons before and during the tonic flexor phase of the “fictive goal-directed motor action” of scratching, under the following working hypotheses. First, spinal neurons exhibit an ON-OFF and OFF-ON firing activity as the neurons from the obex region (Tapia et al., 2013). Second, the fictive “postural stage” of the hindlimb during fictive scratching is just associated with the neuronal tonic activity of the OFF-ON spinal neurons. We found data supporting these hypotheses by using a larger time window for observation than the time window of a scratching episode.

## Methods

### Surgery

Surgical procedures have been described in detail previously (Cuellar et al., 2009). Experimental procedures strictly observed the guidelines of the European Communities Council Directive of 24-November-1986 (86/609/EEC), National Institutes of Health Guide for the Care and Use of Laboratory Animals (85–23, revised in 1985) and the Mexican regulations (NOM-062-ZOO-1999). The protocol was approved by the ethics committee (CICUAL-Proyecto-00489) from the Benemérita Universidad Autónoma de Puebla. Briefly, data were obtained from 13 adult cats with a weight of 2.0–3.5 kg. Animals were anesthetized with isoflurane at 2%. A peripheral vein was catheterized to administer solutions and drugs during the experiment. Arterial blood pressure was monitored and kept above 80 mmHg. In case of blood pressure drop, dextran solution was administered. Laminectomies were performed in L4-S1, and C1-C2 spinal segments and dura mater was cut. The flexor nerve Tibialis anterior (TA) and the extensors lateral gastrocnemius plus soleus (LGS) and medial gastrocnemius (MG) were dissected bilaterally and used for electrophysiological recordings. After craniotomy, a precollicular-postmammillary decerebration was performed and anesthesia discontinued. Right after, pancuronium bromide (Pavulon; Organon) was administered, and artificial ventilation was provided. A heating pad and radiant heat lamp were used to keep the temperature of the animals close to 37°C.

### Electrophysiological recording

We applied D-tubocurarine (0.1%, 14 mM) on the C1–C2 segments to induce fictive scratching after the mechanical stimulation of the pinna or adjacent receptive fields. Electrical field potentials of the spinal cord were recorded in AC or DC mode (Synamps EEG amplifier, NeuroScan) using a multielectrode array composed of 30 Ag–AgCl electrodes (200 μm diameter rounded-tip). The array was placed on the dorsal surface of L4–S1 (for further details see Manjarrez et al., 2005; Cuellar et al., 2009). Electrical field potentials recorded with the multielectrode array were used to create topographic maps (Scan 4.2 software, NeuroScan). The spinal cord segment with the highest amplitudes in both positive DC-shift and the sinusoidal-CDPs was identified. Maximal amplitudes occurred around L6 spinal cord segment (data not shown); therefore micropipette insertions were performed at this spinal segment. Intraspinal single unit activity and electroneurographic (ENG) activity (0.05 Hz to 30 kHz band-pass) was recorded using AC amplifiers (Grass P511) and the Digidata system (Molecular Devices). Sampling frequency was 10 kHz. Simultaneously, electrical field potentials were recorded on the surface of the spinal cord by means of a silver ball electrode (Ag–AgCl), placed in the same segment of the single intraspinal unit recordings site. Glass micropipettes (7–15 MΩ) filled with NaCl (1.2 M) were used to record the extracellular single unit activity of spinal neurons. In other series of experiments, we used quartz/platinum–tungsten fiber electrodes for the multiunit neuronal activity (impedance 5–7 MOhms) with the Minimatrix system (Thomas Recording). Neurons were recorded in the deep dorsal horn, intermediate nuclei and ventral horn of the L6 segment of the spinal cord.

### Spike sorting and spike rate histograms

We obtained recordings of spinal neurons and analyzed the peri-event neuronal discharge of neurons from the lumbar spinal cord (*n* = 95) before, during and after the tonic flexor phase of fictive scratching. Such tonic flexor phase can be obtained from the activity of the TA flexor nerve. Neuronal firing activity was classified according to Berkinblit et al. (1978a,b) into three groups: extensor, intermediate, and flexor neurons, according to their discharge in the scratching cycle and the TA and GM nerves. We employed the unsupervised spike-sorting algorithm “wave-clus” developed by Quiroga et al. (2004) to select the unitary spikes from the recordings.

Spike rate histograms around the tonic flexor phase were constructed using raster displays analyzed for each firing activity of the rhythmic neurons displaying action potentials during a time window equal to the duration of the tonic phase. We normalized such windows before and after the beginning of the tonic flexor phase (from −100 to +100%), taking as a reference (zero time) the beginning of the discharge of the TA nerve.

### Circular statistics

We determined the rhythmicity phase of the neurons through the statistical analysis of the firing activity of the neurons employing circular statistics and the Rayleigh test (*p* < 0.05). Because the cycle of rhythmical activity of the interneurons occurs during the scratching cycle, it is possible to consider the interneuron-activity cycle as a circle, with 0° defined as the onset of the TA nerve activity and 180° defined as the offset of TA nerve activity. Each value of the mean neuronal firing discharge per bin was considered as the length of a vector. Finally, the angle of this vector was defined as the angle of the center of the bin within the cycle. The firing activity of individual neurons during 10 consecutive scratching cycles was analyzed using circular statistics. With this method we obtained a vector indicating the phase preference of such rhythmical discharge (see Mardia, 1972; Batschelet, 1981; Berkowitz and Stein, 1994; Cuellar et al., 2014). A mean vector of length “0” indicates a random mean firing rate consequently not associated with the cycle of the TA nerve activity. Conversely, a mean vector of the length of 1 indicates that all the neuronal discharge occurred within the same bin in each cycle.

### Statistical analysis

The statistical differences between the median of frequency distribution before vs. during tonic flexor phase were determined by the non-parametric Signed-Rank test. The medians were considered significantly different when *p* < 0.001. Histograms of the mean firing rate across the population of neurons were obtained from the mean instantaneous frequency as a function of time. The instantaneous firing rate corresponded to the inverse of inter-spike intervals.

## Results

**Figure 1:**
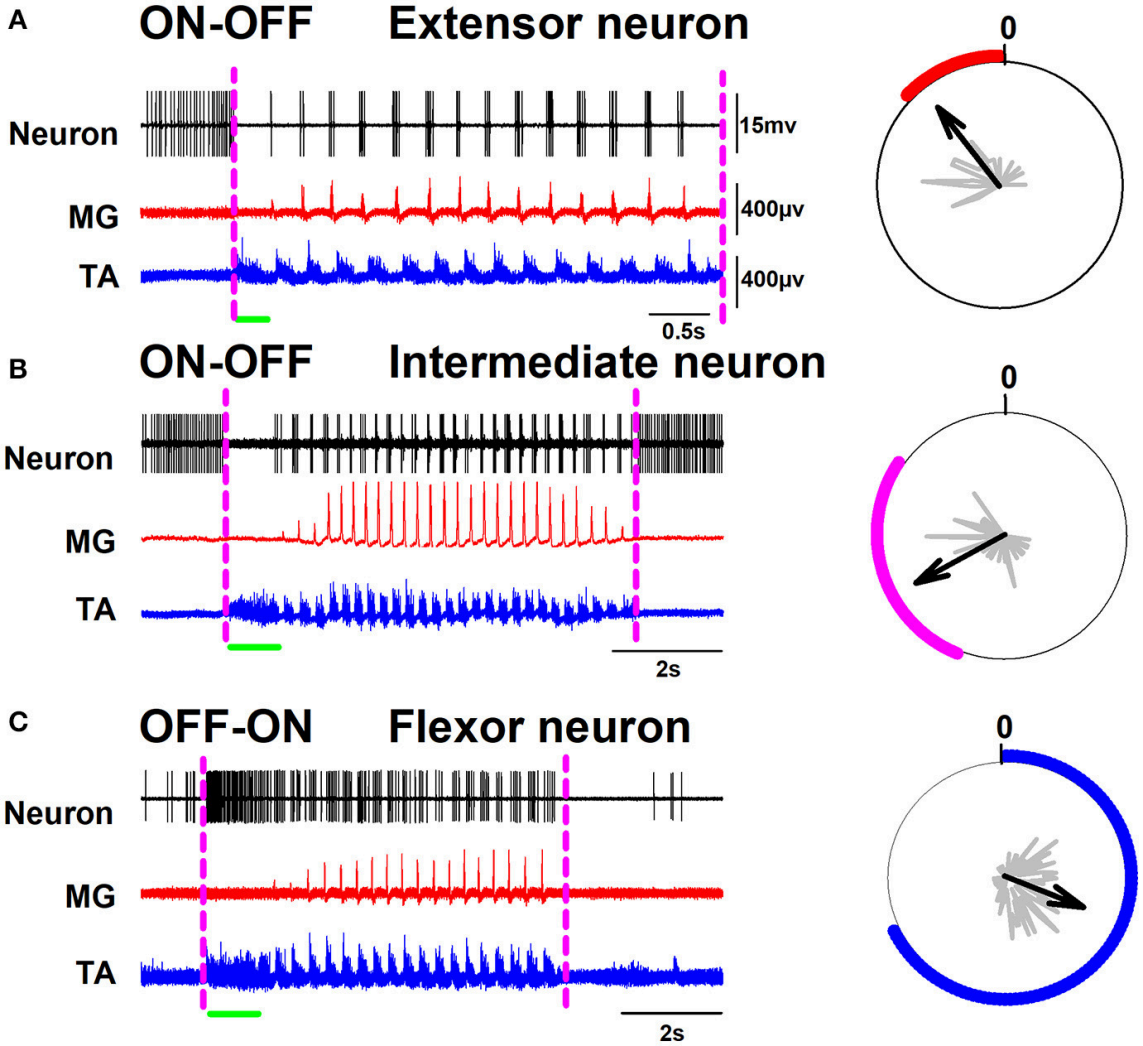
Representative spinal neurons are exhibiting ON-OFF and OFF-ON firing activity during scratching. **(A)** Left panel, recordings of an extensor neuron and the electroneurograms of the medial gastrocnemius (MG) and tibial anterior (TA) nerves, Right panel, a polar plot of the neuronal firing activity. **(B,C)** the same as **(A)** but for an intermediate and a flexor neuron, respectively. The green horizontal line indicates the tonic flexor phase in the electrical activity of the TA nerve.

**Figure 2:**
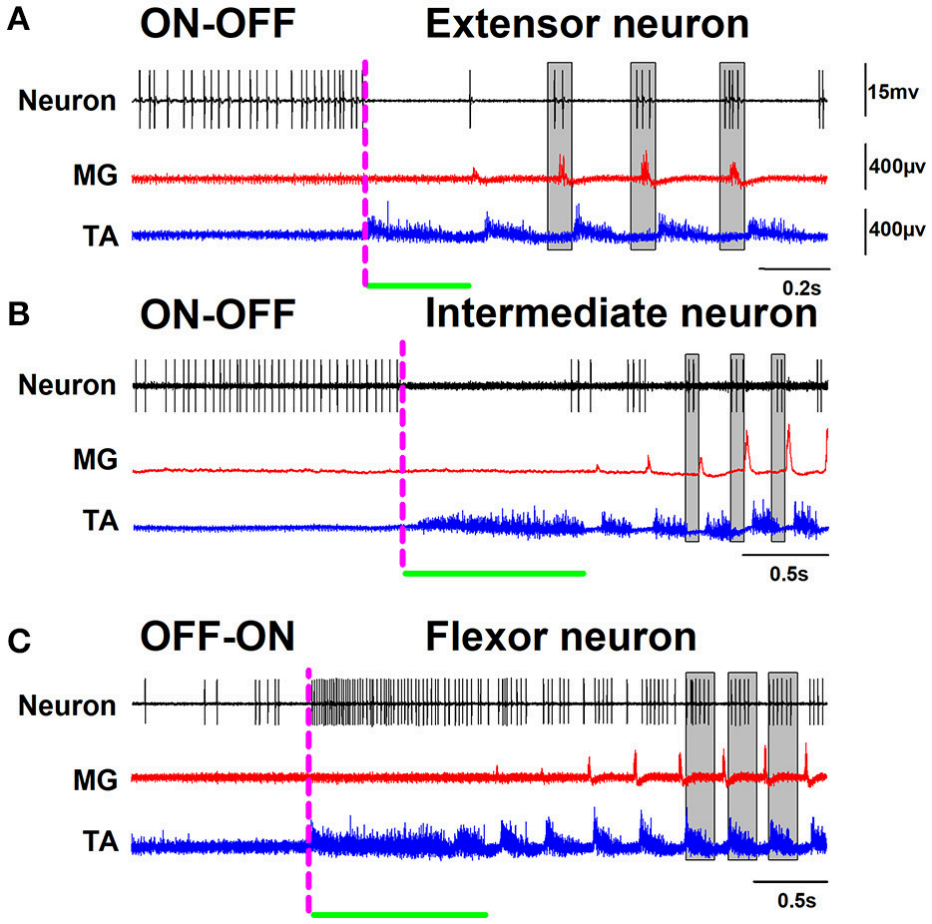
The same as Figure 1 but on another timescale. The gray rectangles highlight in detail the phase relationship of the extensor, intermediate, and flexor neurons to the nerve activity.

A surface multielectrode-array was used to record the positive DC-shift and sinusoidal-CDPs during scratching. Then, the spinal segments with the highest amplitudes were identified, and the extracellular unitary recordings were performed as described previously (Cuellar et al., 2009, 2014; Tapia et al., 2013). We obtained intraspinal recordings in a time window larger than the scratching episodes. The left panel of Figure 1 illustrates the activity of representative extensor, intermediate, and flexor neurons in such time window. The right panel in Figure 1 shows polar plots of the firing activity, employed to verify the firing phase of each neuron. The dashed magenta lines in Figure 1 indicate the beginning and end of the scratching episode. The first trace in Figures 1A–C, corresponds to the single unit activity of an extensor, intermediate, or flexor neuron, respectively. The second trace in red color shows the extensor MG activity. The third trace in blue color is the flexor TA activity. Observe that this activity of TA has a tonic flexor phase (indicated by the green horizontal line). Note the alternation between the bursting activity of TA and MG. In Figures 1A,B, background activity of the neuron is eliminated during the entire tonic phase. Then the neuronal discharge becomes rhythmical and is associated with the activity of the nerves (extensor neuron: see red polar plot on the right panel of Figure 1A, and intermediate neuron: see magenta polar plot on the right panel of Figure 1B). Note that the firing rates of the extensor and intermediate neurons, illustrated in Figures 1A,B, change from a sustained firing activity to an absence of firing activity during the tonic flexor phase. After this absence of activity, the extensor neurons follow the rhythmic activity of MG while the intermediate neuron exhibits activity at the end of the flexor bursts and before the activity of MG (see polar plots on the right panels). We observed that the sustained firing activity of both extensor and intermediate neurons returns after the end of the scratching episode. Moreover, we would like to comment that we observed a slow ramping up of spike frequency well before the initiation of the tonic activity of TA (posture) (see Figure 1). Figure 2B shows in more detail the phase relationship of the intermediate neuron to the MG activity. We defined this firing activity: ON-OFF. In contrast, the flexor neurons exhibited the opposite activity as shown in Figure 1C. Observe the low firing activity of such neuron before the beginning of the scratching episode, which exhibits a dramatic frequency increase during the tonic phase of the flexor TA activity. After this tonic phase, the firing activity of the neuron becomes rhythmic at the same phase of the TA cycles (see blue polar plot on the right panel of Figure 1C). Figure 2C shows in more detail the phase relationship of the intermediate neuron to the TA activity. We termed this firing activity as OFF-ON.

**Figure 3:**
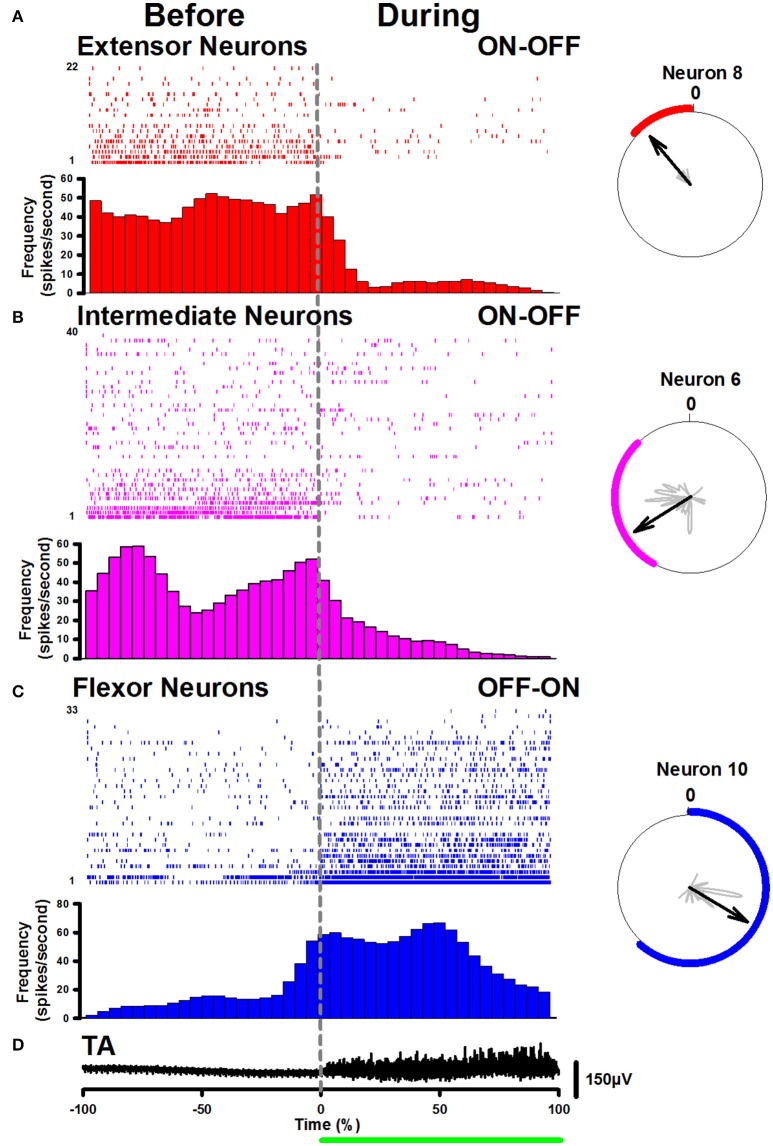
Peri-event firing rates of spinal interneurons before and during the tonic flexor phase. **(A)** Raster display of 22 rhythmical neurons classified as extensor (red traces). **(B)** Raster display of 40 rhythmical neurons classified as intermediate (purple traces). **(C)** Raster display of 33 rhythmical neurons classified as flexor (blue traces). The beginning of the tonic flexor phase is indicated by the vertical dashed line (zero time as reference). **(D)** Electroneurogram of the tonic flexor phase of the TA nerve (black). The green horizontal line indicates the tonic flexor phase in the electrical activity of the TA nerve. The alternating rhythmical activity was excluded from the raster displays for simplicity. The corresponding histograms are illustrated below the raster plots. The histograms illustrate the mean instantaneous frequency as a function of time across the population of recorded neurons. The polar plots of some neurons are illustrated in the right panel. The polar plots show the phase of the interneuronal firing activity relative to the cycle of the TA nerve.

To analyze in detail the ON-OFF and OFF-ON firing activity of these neurons, corresponding raster plots around the tonic phase of the flexor TA activity were pooled (Figure 3). Figure 3D shows a representative tonic flexor phase of the TA activity. The duration of the tonic flexor phase for all the scratching episodes was normalized from −100 to +100%. The raster plots were classified according to its respective polar plot. For example, if the polar plot of a raster plot was in phase with the extensor rhythmical MG activity, then we placed such raster plot in Figure 3A. If the polar plot was in phase with the intermediate activity, then we placed the raster plot in Figure 3B. Finally, when the polar plot was in phase with the flexor TA rhythmical activity, we placed the raster plot in Figure 3C. In general, the polar plots were used to quantify statistical differences in the phase relationship of the extensor, intermediate and flexor neurons to the nerve activity. Once we identified the rhythmic phase of these neurons, we plotted the raster plots around the tonic flexor TA-activity. The polar plots dictated which neurons were extensor, flexor and intermediate. Then the subsequent observation of the raster plots allowed an objective classification in ON-OFF or OFF-ON patterns of the spinal neurons. We recorded 22 extensor neurons with an ON-OFF firing activity as illustrated in Figure 3A. The sustained median frequency of discharge of these neurons was significantly reduced from 45.7 to 5.8 spikes/s, around the beginning of the scratching episode. Furthermore, we recorded 40 intermediate neurons with a similar ON-OFF firing activity as illustrated in Figure 3B. The sustained median frequency of firing was also significantly reduced from 40.7 to 8.9 spikes/s, around the beginning of the scratching episode. Moreover, we recorded 33 flexor neurons with an OFF-ON firing activity as illustrated in Figure 3C. The low median frequency of firing was significantly increased from 13.4 to 53.7 spikes/s, around the beginning of the scratching episode. Table 1 shows the statistical analysis for these comparisons.

**Table 1:** We performed the Signed-Rank test to compare the median of the firing rate before and during scratching episodes.

**Signed-rank Test**						
**Before vs. During**						
	**Median firing rate before (spikes/s)**	**Median firing rate during (spikes/s)**	***T***	***z***	***r***	***p***
**Extensor neurons**	45.7	5.8	0.0	−3.92	−0.88	0.00008
**Intermediate neurons**	40.7	8.9	1.0	−3.88	−0.87	0.0001
**Flexor neurons**	13.4	53.7	3.33	−3.55	−0.79	0.0003

The comparisons showed highly significant differences between firing rates before vs. firing rates during scratching (^***^P < 0.001) in extensor, intermediate and flexor neurons, respectively.

**Figure 4:**
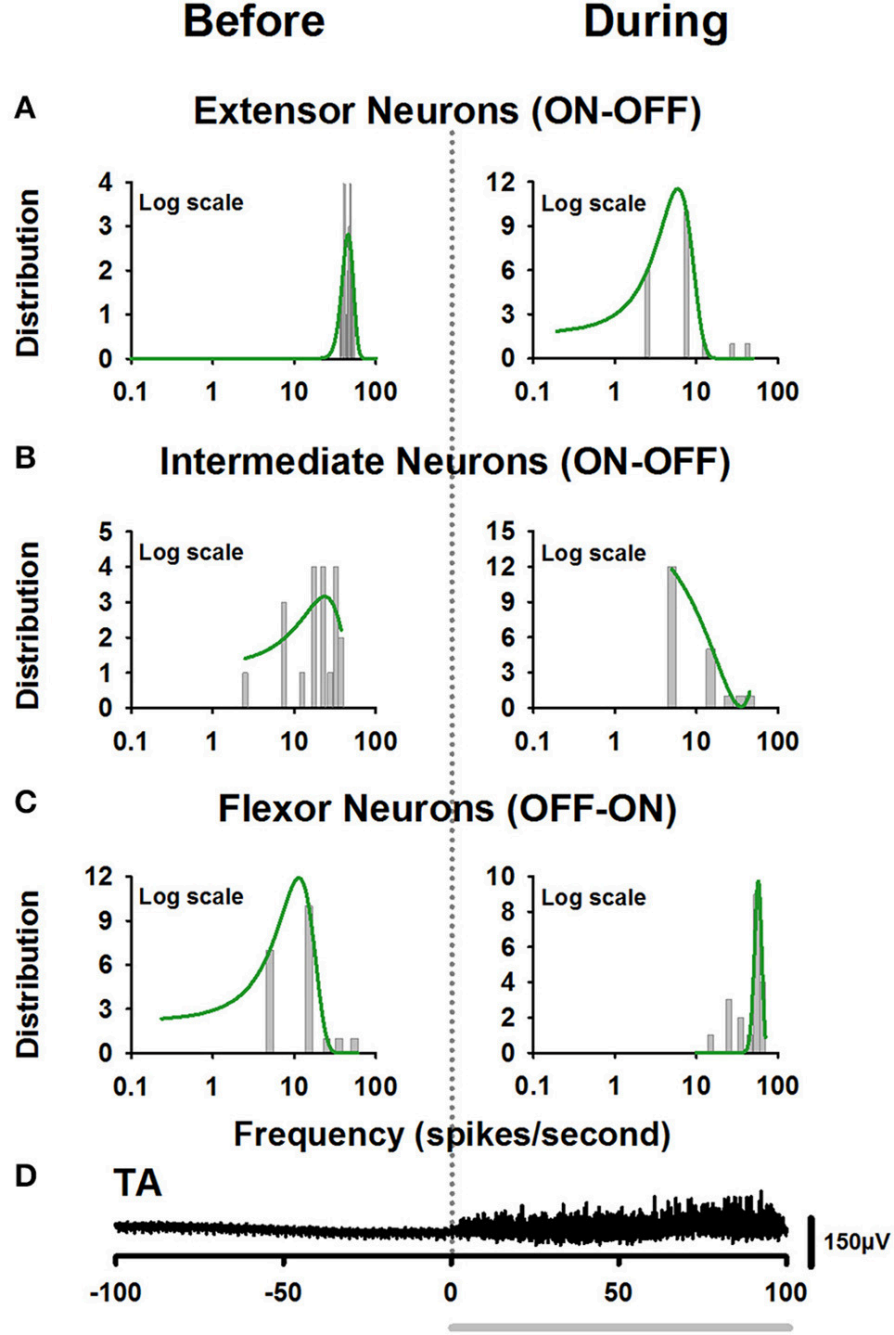
Firing rate distributions obtained from the firing activity illustrated in Figure 3. **(A)** Left panel, lognormal distribution of firing rate of extensor neurons before the onset of fictive scratching. The right panel shows the skewed lognormal distribution of firing rate, for the same extensor neurons after the onset of fictive scratching. **(B)** The same as Figure **(A)**, but for intermediate neurons. Note that these neurons did not exhibit a complete lognormal-firing-rate distribution. **(C)** The same as **(A)**, but for the flexor neurons. **(D)** Electroneurogram of the tonic flexor phase of the TA nerve (black). The gray horizontal line indicates the tonic flexor phase in the electrical activity of the TA nerve.

In a recent study, Petersen and Berg (2016) demonstrated that the firing rate of populations of neurons from the turtle spinal cord is normally distributed on a log-scale (lognormal) (see also Berg, 2017). We examined whether the firing rate before and after the onset of movement for the extensor, intermediate, and flexor interneurons is also lognormally distributed. We found that only the firing rate of extensor and flexor neurons is lognormally distributed. The intermediate neurons did not exhibit a complete lognormal-firing-rate distribution (Figure 4). Table 2 shows the Gaussian equations and the fitting parameters.

**Table 2:** Gaussian equations and fitting parameters for the distributions illustrated in Figure 4.

	**Before**			**During**		
	**Equation**	**Parameters**		**Equation**	**Parameters**	
Extensor neurons	*f* = *a* ^*^ exp(−0.5 ^*^ ((*x* – *x*_0_/*b*)^∧2^)	*a*	2.82	*f* = *a* ^*^ exp(−0.5 ^*^ ((*x* – *x*_0_/*b*)^∧2^)	*a*	11.53
		*b*	7.21		*b*	2.98
		*x_0_*	45.68		*x_0_*	5.9
Intermediate neurons	*f* = *a* ^*^ exp(−0.5 ^*^ ((*x* – *x*_0_/*b*)^∧2^)	*a*	3.15	*f* = *y*_0_ + *a* ^*^ exp(−0.5^*^((*x* – *x*_0_/*b*)^∧2^)	*a*	−50274.5
		*b*	16.54		*b*	1398.16
		*x_0_*	23.55		*x_0_*	35.11
					*y_0_*	50274.64
Flexor neurons	*f* = *a* ^*^ exp(−0.5 ^*^ ((*x* – *x*_0_/*b*)^∧2^)	*a*	11.91	*f* = *a* ^*^ exp(−0.5 ^*^ ((*x* – *x*_0_/*b*)^∧2^)	*a*	9.71
		*b*	6.15		*b*	5.8
		*x_0_*	11.35		*x_0_*	57.27

**Figure 5:**
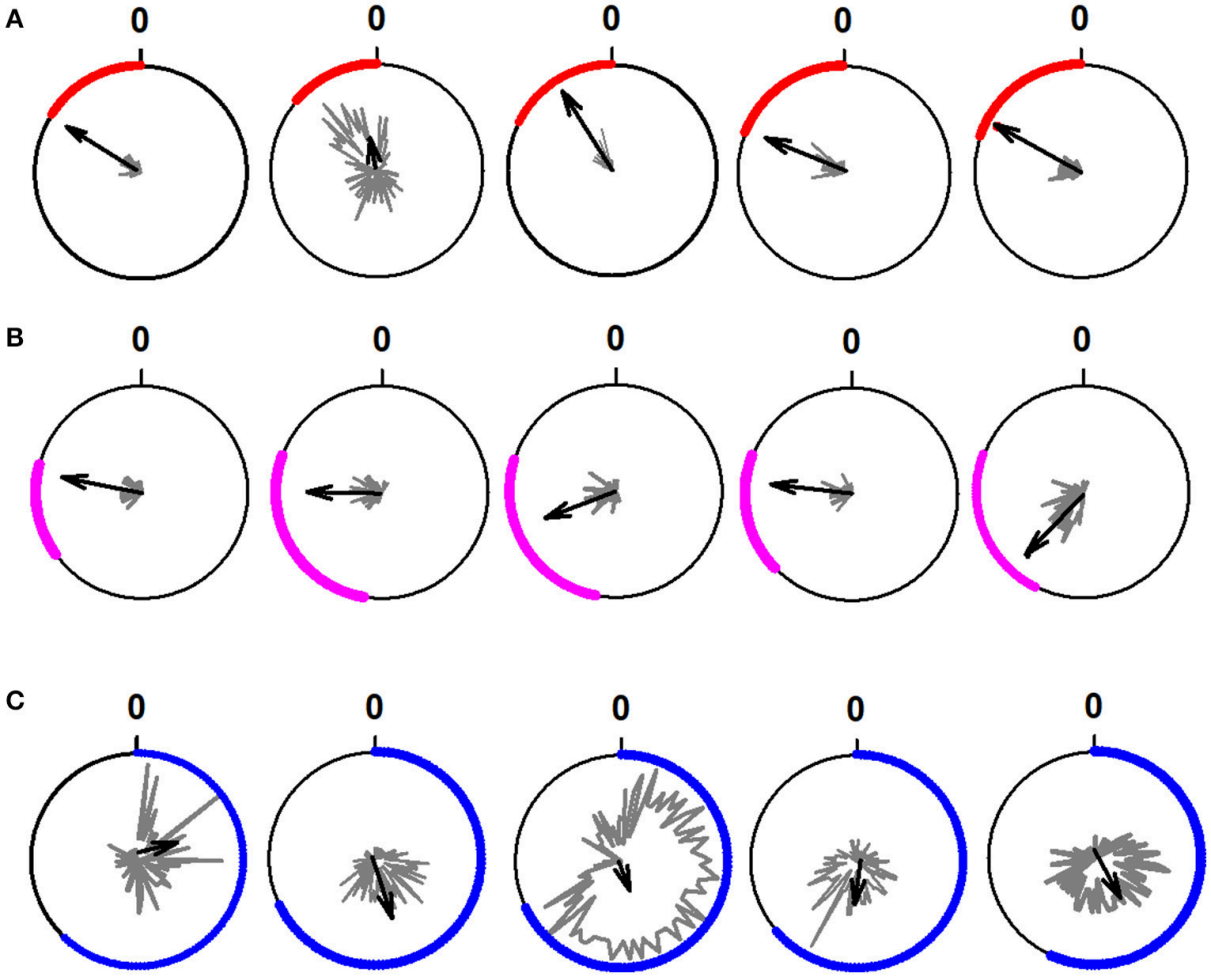
Examples of polar plots obtained from the rhythmical firing activity of 15 neurons during the scratching cycle. **(A)** Polar plots for five extensor neurons. **(B)** Polar plots for five intermediate neurons. **(C)** Polar plots for five flexor neurons. We determined the rhythmicity of the neurons through the statistical analysis of the firing activity of the neurons employing circular statistics and the Rayleigh test (*p* < 0.05). The polar plots show the phase of the interneuronal firing activity relative to the cycle of the TA nerve. The length of the black arrows represents the mean neuronal firing discharge. The direction of the black arrows indicates the phase preference of the rhythmical discharge.

In Figure 5, we show 15 typical examples of polar plots, which were used to classify the raster plots illustrated in Figure 3. We obtained the polar plots from the rhythmical activity of all the 95 spinal neurons.

With this pooling, it is noticeable that the “extensor and intermediate” vs. “flexor” neurons have opposite firing activities, i.e., ON-OFF vs. OFF-ON. We compared the spike rate histograms of the extensor and intermediate neurons with the spike rate histogram of the flexor neurons. We performed statistical analyses based on the firing frequency “before” vs. “during” the tonic flexor phase for the flexor, extensor, and intermediate neurons. Statistically significant differences (Mann-Whitney test, *p* < 0.001) in the mean firing frequency before vs. during the tonic phase were found in the three groups.

## Discussion

We analyzed perievent firing rates of spinal neurons before and during the fictive goal-directed motor action of scratching in decerebrate cats. We found that the “extensor and intermediate” neurons exhibit opposite firing activity compared to “flexor” neurons during fictive scratching, i.e., ON-OFF vs. OFF-ON. These firing activities are similar to those of neurons from the reticular formation in the brainstem at the level of the obex during scratching (Tapia et al., 2013). This comparison suggests a possible functional relationship between ON-OFF and OFF-ON spinal neurons and the ON-OFF and OFF-ON neurons from the obex reticular formation described in a previous study from our laboratory (Tapia et al., 2013). In the study by Tapia et al. (2013), we found that the ON-OFF and OFF-ON neurons also exhibited this firing activity associated with the scratching episodes. Furthermore, we found an obex slow potential (i.e., a DC-shift) which was correlated to the DC-shift that previously we reported during the scratching episodes (Cuellar et al., 2009). Future experiments, with simultaneous recordings from the obex reticular formation and the lumbar spinal cord, will be necessary to examine the functional link between these supraspinal and spinal neuronal groups.

In the present study, we selected the L6 spinal cord segment to perform single unit recordings because in this segment the scratching CPG neurons exhibit the largest sinusoidal-CDPs (Cuellar et al., 2009). Furthermore, in this segment, there are neurons with rhythmical bursting activity in synchrony with scratching (Berkinblit et al., 1978a,b; Baev et al., 1981) are consistent with previous studies by Berkinblit et al. (1978a,b), Baev et al. (1981) and Barajon et al. (1992). Moreover, a broader time window for the recording of the scratching CPG neurons in the L6 segment was used to find the ON-OFF and OFF-ON firing activity of these neurons. In the original classification of the firing discharge of neurons from the lumbosacral spinal cord during scratching, Berkinblit et al. (1978a,b), did not report such ON-OFF and OFF-ON activity. This could be due in part to the relatively short time window employed in the analysis by these authors; a time window mostly focused on the rhythmical part of the scratching reflex and the tonic phase.

Berkowitz (2001) found a population of spinal neurons that tended to be rhythmically active in a specific-phase manner related to the cycle of the hip flexor muscle during all three forms of ipsilateral fictive scratching in the turtle (rostral, pocket and caudal). This finding is consistent with our findings and the original description by Berkinblit et al. (1978a,b), that the rhythmically active spinal neurons in the cat are active in a consistent phase of the flexor TA rhythm during fictive scratching. However, because the cat only exhibits one type of scratching we did not classify preferred phases of scratching as Berkowitz (2001) in the turtle.

The ON-OFF and OFF-ON spinal neurons could receive descending inputs from the brainstem and other supraspinal centers; however, these neurons also could send ascending projections throughout spinocerebellar axons. Two findings support this possibility. The first is that the dorsal and ventral spinocerebellar tracts in the cat receive input from scratching CPG neurons (Fedirchuk et al., 2013). The second is that in the cerebellar vermis of the cat there is a sinusoidal electrical activity as in the lumbar spinal cord during scratching (Martínez-Silva et al., 2014). Based on these reports, we suggest that in the cerebellar vermis also there are groups of neurons exhibiting ON-OFF and OFF-ON firing activity, very similar to those found in the present study for the lumbar spinal neurons during scratching.

The firing activity of descending spinal axons has also been classified according to muscle activation during the scratching repertoire in the turtle (Berkowitz and Stein, 1994). This link between descending actions and spinal circuits is consistent with our findings that the ON-OFF and OFF-ON firing activity of spinal neurons are similar to the firing of neurons from the brainstem, also ON-OFF and OFF-ON. However, Currie and Stein (1990) demonstrated that the cutaneous stimulation in the turtle produces long-lasting activation of spinal neurons after complete transection at the forelimb enlargement. This suggests that spinal neurons have a segmental repertoire to increase the excitability of spinal neurons even without descending inputs. However, additional information about the firing activity before and after fictive motor patterns in this animal model is still missing. Similarly, Geertsen et al. (2011) provided valuable information about the inhibitory activity of spinal neurons leading to the generation of alternation between antagonistic muscles during fictive locomotion and scratching in the cat. They reaffirmed the notion that the spinal circuits are responsible for producing a goal-directed motor action and integrating sensory information in the absence of supraspinal influences, although this information was provided solely for the rhythmic alternation in recorded muscles.

As mentioned above, Tapia et al. (2013) described two types of bulbar neurons (ON-OFF and OFF-ON) presumably involved in the triggering (i.e., activation) of the CPG, because their activity began prior the scratching episode. Tapia et al. (2013) suggested that the ON-OFF and OFF-ON bulbar neurons could be part of the reticular activating system (RAS), necessary to initiate the motor activity. In this context, it will be necessary for future experiments, to obtain simultaneous recordings from the reticular formation and the spinal cord before and during scratching. These experiments could be relevant to examine the identity of spinal neurons that receive the tonic drive that ultimately activates the CPG. A possible experimental paradigm could be the simultaneous recording of “ON-OFF,” “OFF-ON,” and “tonic” neurons in the spinal cord and the “obex reticular formation” during scratching. It is possible that we could identify the “tonic” neurons in both regions: the spinal cord and the obex reticular formation. The identity of these tonic neurons could be clarified using the stimulation of several nerves to examine the afferent inputs to these “tonic” neurons. Such study could also be interesting because there is evidence that firing neurons with tonic activity are essential even in goal-directed motor actions executed automatically (see Apicella et al., 1991, 1997, 2011; Ravel et al., 2001).

The hypothesis that the goal-directed motor action is associated with the neuronal tonic activity is consistent with the functional mechanism that explains the spinal firing activity during the scratching motor task. The scratching starts when the whole active hindlimb reaches the surface of the skin (Sherrington, 1910; Kuhta and Smith, 1990), with a characteristic “postural stage” of sustained flexion. Our results illustrated in Figure 1C and Figure 3C are consistent with the hypothesis that the fictive goal-directed motor action to maintain the fictive “postural stage” of the hindlimb during fictive scratching is associated with the neuronal tonic activity of the OFF-ON spinal neurons. In general, this tonic phase is characterized by the tonic activation of flexor nerves (Deliagina et al., 1983).

The “rhythmical stage” of scratching is performed after the “postural stage,” when the hindlimb carries out rhythmic movements of alternation between flexor and extensor muscles. We found that during this rhythmic activity of the motoneurons the ON-OFF spinal neurons are in synchrony with the extensor or intermediate phases of the fictive scratching cycle (Figures 1A,B, 2A,B, 3A,B, 5A,B). We observed that when the fictive “rhythmical stage” ends, the sustained firing-activity of the ON-OFF spinal neurons becomes tonic again, as before the fictive scratching episode (see Figure 1B). This observation was consistent with all the recorded ON-OFF spinal neurons. This suggests that the fictive goal-directed motor action of a scratching episode is associated with the suppression of the tonic activity of ON-OFF spinal neurons. The functional meaning of the return of this sustained firing activity of extensor (and intermediate) neurons after the end of the fictive rhythmical stage suggests that in the spinal cord there are neurons providing a continuous extensor tone to the extensor muscles.

Conversely, the OFF-ON spinal neurons are in synchrony with the flexor phase of the scratching cycle (Figures 1C, 2C, 3C, 5C). These neurons only exhibit tonic activity during the tonic phase of the fictive “postural stage” of sustained flexion. It means that before and after the scratching episode the flexor OFF-ON spinal neurons do not exhibit tonic firing activity. The functional meaning of this finding is that in the decerebrate cat there is not a flexor tone in control conditions without scratching. The fictive goal-directed motor action of scratching is associated with an increase in the neuronal tonic activity of flexor neurons only during the fictive “postural stage” of scratching. This is consistent with a previous report by Berkinblit et al. (1980), who found that during the “postural stage” of fictive scratching there is a tonic discharge of the flexor tibial anterior (TA) motoneurons, without discharge during this stage in the extensor gastrocnemius and soleus (GS) motoneurons.

The studies performed by Petersen and Berg (2016) contribute to our understanding of the spinal circuitry responsible for scratching in a new perspective. These authors demonstrated for the first time that the behavior of neural populations in the spinal cord could not be properly described by the mean and standard deviation of their firing rate (Berg, 2017). They found that the firing rate of spinal neurons during different forms of scratching is lognormally distributed (Petersen and Berg, 2016). We found similar results for the extensor and flexor neurons, which exhibit specific lognormal distributions of firing rates (see Figures 4A,C and Table 2). However, we found that the intermediate neurons exhibit a less organized and incomplete lognormal distribution (see Figure 4B and Table 2). This difference supports the hypothesis that the intermediate neurons are a distinct class of spinal neurons involved in the CPG. On the other hand, it is interesting that the transition of firing activity of the extensor ON-OFF neurons around the onset of fictive movement changes from a lognormal distribution to a skewed lognormal distribution. In contrast, the opposite occurs for the flexor OFF-ON neurons (see Figures 4A,C). This suggests that both groups of neurons share similar mechanisms for a balanced ON-OFF and OFF-ON behavior around the beginning of the scratching task.

We conclude that an episode of fictive goal-directed motor action of scratching involves the suppression of tonic activity of spinal (extensor and intermediate) neurons and the concomitant increase of activity of flexor neurons during the fictive “postural stage.” These findings could be relevant for future experiments to understand the activation of spinal CPGs, traditionally studied during rhythmic stages.

## Author contributions

EM: Conceived, designed the experiments and wrote the manuscript; CC, NH, and EM: Performed the experiments; CC, NH, BD, RD-L, RO-K, and EM: Performed the analysis and wrote the Discussion. All the authors revised and approved the manuscript.

### Conflict of interest statement

The authors declare that the research was conducted in the absence of any commercial or financial relationships that could be construed as a potential conflict of interest.
